# Image Thresholding Improves 3-Dimensional Convolutional Neural Network Diagnosis of Different Acute Brain Hemorrhages on Computed Tomography Scans

**DOI:** 10.3390/s19092167

**Published:** 2019-05-10

**Authors:** Justin Ker, Satya P. Singh, Yeqi Bai, Jai Rao, Tchoyoson Lim, Lipo Wang

**Affiliations:** 1 Neurosurgery, National Neuroscience Institute, Singapore 308433, Singapore; justinker@gmail.com (J.K.); jai.rao@singhealth.com.sg (J.R.); 2 School of Electrical and Electronic Engineering, Nanyang Technological University, Singapore 639798, Singapore; satya@ntu.edu.sg (S.P.S.); BA0001QI@e.ntu.edu.sg (Y.B.); 3 Neuroradiology, National Neuroscience Institute, Singapore 308433, Singapore; tchoyoson.lim@singhealth.com.sg

**Keywords:** 3D convolutional neural networks, machine learning, CT brain, brain hemorrhage

## Abstract

Intracranial hemorrhage is a medical emergency that requires urgent diagnosis and immediate treatment to improve patient outcome. Machine learning algorithms can be used to perform medical image classification and assist clinicians in diagnosing radiological scans. In this paper, we apply 3-dimensional convolutional neural networks (3D CNN) to classify computed tomography (CT) brain scans into normal scans (N) and abnormal scans containing subarachnoid hemorrhage (SAH), intraparenchymal hemorrhage (IPH), acute subdural hemorrhage (ASDH) and brain polytrauma hemorrhage (BPH). The dataset used consists of 399 volumetric CT brain images representing approximately 12,000 images from the National Neuroscience Institute, Singapore. We used a 3D CNN to perform both 2-class (normal versus a specific abnormal class) and 4-class classification (between normal, SAH, IPH, ASDH). We apply image thresholding at the image pre-processing step, that improves 3D CNN classification accuracy and performance by accentuating the pixel intensities that contribute most to feature discrimination. For 2-class classification, the F1 scores for various pairs of medical diagnoses ranged from 0.706 to 0.902 without thresholding. With thresholding implemented, the F1 scores improved and ranged from 0.919 to 0.952. Our results are comparable to, and in some cases, exceed the results published in other work applying 3D CNN to CT or magnetic resonance imaging (MRI) brain scan classification. This work represents a direct application of a 3D CNN to a real hospital scenario involving a medically emergent CT brain diagnosis.

## 1. Introduction

Intracranial hemorrhage is a medical emergency that can have high morbidity and mortality if not diagnosed and treated immediately. This condition affects 40,000 to 67,000 patients in the United States annually and up to 52% of patients die within one month [1]. Three commonly-encountered sub-types of intracranial hemorrhage are subarachnoid hemorrhage (SAH), intraparenchymal hemorrhage (IPH), acute subdural hemorrhage (ASDH). In a severe brain trauma, various permutations of SAH, IPH, and ASDH can be seen, which we have termed brain polytrauma hemorrhage (BPH) in this work. The common causes of SAH are trauma and cerebral aneurysmal rupture, while IPH can be caused by hypertension, amyloid angiopathy, brain tumor hemorrhage or trauma. ASDH and BPH appear because of head trauma.

When patients with intracranial hemorrhage present to the emergency department, a computed tomography (CT) scan of the brain is done to diagnosis intracranial hemorrhage, so that medical or surgical treatment can follow. CT scans work by exploiting the differential absorptive properties of body tissues placed between an x-ray emitter and detector. Brain tissue, blood, muscle, and bone give rise to different levels of x-ray attenuation, expressed in Hounsfield units. By moving the x-ray emitter and detector circumferentially around a subject, a three-dimensional image of the subject’s internal tissues is obtained.

**Figure 1:**
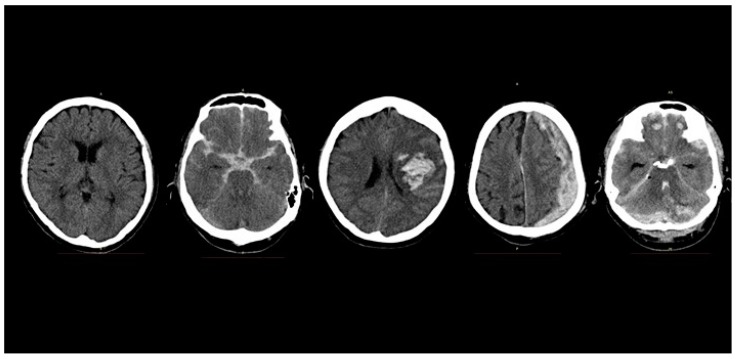
Computed tomography (CT) brain scans. From left, normal (N), subarachnoid hemorrhage (SAH), intraparenchymal hemorrhage (IPH), acute subdural hemorrhage (ASDH), brain polytrauma hemorrhage (BPH). Each image represents an individual image slice. One patient’s complete stack of CT images contained between 24 to 34 image slices in our dataset.

Limitations in the availability or experience of clinicians, especially in rural or resource-strapped health systems, to diagnose CT brains quickly can cause treatment delays. Automating the diagnosis of CT brain scans, or assisting the clinician in triaging critical from normal scans, would help patients by expediting their treatments and improve outcomes. Figure 1 shows the axial slices of five CT brain scans showing a normal brain, SAH, IPH, ASDH, and BPH. The outer rim of uniform white skull bone surrounds the dark grey brain tissue, and areas of acute hemorrhage, appear as patchy, light-grey areas of varying shapes.

The artificial neuron was first described by McCulloch and Pitts in 1943 [2]. This has evolved through the symbolic, rule-based artificial intelligence (AI) paradigms, to manual feature-handcrafting algorithms, and to modern multi-layered or “deep” neural networks, which perform feature detection and classification automatically. Convolutional neural networks (CNN) owe their inception to Fukushima’s Neocognitron model in 1982 [3], and their popularity to Lecun et al. [4] and Krizhevsky et al. [5] The latter employed a CNN to win the 2012 Imagenet Large Scale Visual Recognition Challenge, and since then CNNs have been used for many image classification tasks. The advantage of CNNs in image classification is the ability to perform feature-extraction and learn high-level image features automatically without feature-handcrafting, leading CNNs to become the dominant machine learning architecture in image recognition tasks. CNNs have been widely used in machine vision to perform a variety of tasks, such as image classification, object detection, and semantic segmentation. In the medical image analysis space, CNNs have been used for the classification and diagnosis of 2-dimensional medical images, such as chest x-rays, retinal photographs, skin dermoscopic images, and histology images, with performance comparable to or exceeding human clinicians [6,7,8,9].

In analyzing volumetric magnetic resonance imaging (MRI) or CT data, various machine learning approaches including 2D CNN have been attempted. These efforts have involved manual slice selection, extensive manual pre-processing, feature hand-crafting, and segmentation, before classification [10,11,12]. A number of these approaches classify individual images from a volumetric image stack one image at a time, turning a 3D classification problem into a 2D task. Other authors have used employed a combination of 2D CNN in the three planes (axial, coronal, sagittal) that define a 3D volume [13,14,15]. Roth et al. [14] detected abnormal lymph nodes on thoracic CT scans, by decomposing a 3D volume of interest into re-sampled 2D views, and then training their CNN on augmented variations of these 2D views. Their CNN achieved a satisfactory sensitivity of 90%, at six false positives per patient. The disadvantages of these previous strategies include the manual time and effort in hand-crafting, feature segmentation, and stripping, and the potential loss of spatial contextual information when a 3D volume is analyzed using 2D slices.

Three-dimensional CNNs are an emerging architecture, and have been used mainly in analyzing video or 3-dimensional volumetric medical images. Previously, the use of 3D CNN was limited as it was computationally expensive and lengthy to process 3-dimensional kernels and entire volumes of images. However, more papers on 3D CNN have appeared in the scientific literature with their adoption likely aided by decreasing computational hardware costs. In medical image analysis, 3D CNNs have been applied to detecting abnormalities (tumors, hemorrhage, ischemia) in brain, heart, lung, and liver organs on CT or MRI imaging [16,17,18]. As a measure of the interest in the clinical problem of automatically detecting intracerebral hemorrhage on MRI or CT, there is also a growing number of publications on this topic using 3D CNNs [19,20]. Dou et al. [20] analyzed cerebral microbleeds on MRI brain scans using a two-stage 3D CNN that screened and then detected microbleeds. Their method achieved 93% sensitivity, at a cost of 44% precision and 2.7 false positives per patient.

We propose a 3D CNN that classifies CT brain scans into normal (N), subarachnoid hemorrhage (SAH), intra-parenchymal hemorrhage (IPH), acute subdural hemorrhage (ASDH), and brain polytrauma hemorrhage (BPH).

In this work, we aim to create a 3D CNN that can automatically detect and diagnose SAH, ASDH and IPH on CT brain scans, and distinguish them from normal scans. This work would have direct clinical application in emergency departments and acute-care hospitals worldwide. We propose a simple and fast 3D CNN that is effective and accurate. It is hoped that the straightforward implementation of this 3D CNN will lead to its widespread adoption. Specifically, we modify well-known 2D CNN architectures into 3D CNN. The novel aspects of our work are as follow:

(1) To our knowledge, this is the first demonstration of a 3D CNN on volumetric CT brain data that classifies patient scans into different acute hemorrhagic variants, which impacts subsequent medical treatment. Previous work has been limited to detecting normal versus abnormal scans. We also demonstrate that our network performance gives state-of-the-art results in classification accuracy.

(2) We present a novel image thresholding method optimized for the detection of acute hemorrhage on CT brain scans, which mimics the visual analysis of radiological images by human radiologists. We demonstrate that the application of our method improves the classifier performance.

This paper is organized into the following sections. Section 2 describes our methods, with details on the dataset, network architectures, and training protocols. Section 3 reports our results and experimentation with various network parameters. Section 4 analyzes the impact, limitations and future work stemming from our results. Section 5 summarizes and concludes this paper.

## 2. Methods

### 2.1. 3-Dimensional Convolutional Neural Networks

The feature map of a convolution layer is formed by convolving the feature map of the previous layer with learnable kernels, adding a bias term, and then applying an activation function [21]. Specifically, we can define $h_{k}^{p}$ as the $k^{th}$ feature map of the $p^{th}$ layer, and $h_{j}^{p-1}$ as the $j^{th}$ feature map of the previous layer, p−1. $W_{j,k}^{p}$ is a learnable kernel, and $b^{p}$ is the bias term. σ is the activation function, commonly a rectified linear activation unit (ReLu). This is written as:

(1) $$\begin{array}{c} h_{k}^{p}=\sigma \left(\underset{j}{\sum }h_{j}^{p-1}\times W_{j,k}^{p}+b_{k}^{p}\right) \end{array}$$

A 2-dimensional convolution can be defined for convolving an input image I with a kernel K. Extending the equation for a 2-dimensional convolution [22] into a 3-dimensional convolution, we obtain:

(2) $$\begin{array}{c} S\left(\ell ,m,n\right)=\left(K\times I\right)\left(l,m\;,\;n\right)=\underset{a}{\sum }\underset{b}{\sum }\underset{c}{\sum }I\left(\ell -a,m-b,n-c\right)\;K\left(a,\;b,\;c\right) \end{array}$$

Equation (2) may be expressed as:

(3) $$\begin{array}{c} S\left(\ell ,m\;,n\right)=\underset{a,\;b,c}{\sum }h_{j}^{p-1\;}\left(\ell -a,m-b\;,n-c\right)W_{j,k}^{p}\left(a,\;b,\;c\right)\; \end{array}$$

In Equation (3), $h_{j}^{p-1\;}$ is the $j^{th}$ 3-dimensional feature map of the previous layer p−1, and $W_{j,k}^{p}$ is the 3-dimensional kernel. Substituting Equations (1) and (3), we obtain the equation for $h_{k}^{p}$, which is a 3-dimensional feature map:

(4) $$\begin{array}{c} h_{k}^{p}=\sigma \left(S_{j,k}^{p}+b_{k}^{p}\right) \end{array}$$

These 3-dimensional convolution layers were stacked with max-pooling and fully-connected layers. Kernels were initialized with Gaussian distribution and parameters were tuned with standard stochastic gradient descent and cross-entropy loss minimization.

### 2.2. Image Thresholding to Detect Acute Hemorrhage

The CNN extracts features from the input images, and most of the features are associated with edges, shape, and curves present in the input images. It can also be seen by visualizing the different layers of CNN that the first layer mostly picks up edges present in the images, while the second layer picks up curves, and the third layer picks up shapes. The human eye is similar in that it also observes an image for its constituent edges, curves, and shapes, as suggested by the presence of ocular dominance and orientation columns in the primary visual cortex of the mammalian brain. In detecting anomalies on medical images, a region of interest (ROI) may be subtle or not apparent to visual inspection by either human or CNN. Just as thresholding aids a human radiologist to emphasize possibly abnormal areas, we propose a threshold operator to accentuate individual sharp edges, such that this improves the likelihood of a CNN detecting a feature, and, therefore, performing a correct classification.

To improve the discriminatory ability of our model, we propose a novel thresholding method to detect acute hemorrhage. We build on the work of Zhang et al. [23] who used spatial histograms to detect cars in images. There is a range of pixels intensities which is common in both normal and abnormal CT scans. Therefore, we can discard these intensities before feeding the image to the CNN without any loss of information. This is similar to the dimensionality reduction process. In our proposed method, we generate the average pixel intensity histograms of 2 classes of CT scans (such as Normal and SAH), to overlap and search for an optimal pixel intensity threshold that accentuates their difference. Pixel intensities below this threshold are then disregarded. This process creates sharp edges and shapes around a ROI which helps CNN in feature extraction. An added benefit is that a CNN will require less time for training.

The intuition underlying this approach is two-fold. First, we observed that normal CT brain scans, even across different patients are largely homogeneous, which makes a histogram representation of the class meaningful. Abnormal scans can be thought of as the addition of extraneous blood signal to normal scans. Second, we also observed that radiologists adjust image contrast levels when reading CT scans, to accentuate subtle amounts of blood, and to downplay the appearance of normal surrounding tissue. Our method attempts to model this behavior in human visual cognition. Using our method improves the overall performance of the classifier across all classes of acute brain hemorrhage.

### 2.3. Dataset and Pre-Processing

The dataset consists of non-contrast CT brain images from the National Neuroscience Institute at Tan Tock Seng Hospital, Singapore. After institutional review board approval, a search of electronic discharge summaries with the diagnoses matching head injury or intracranial hemorrhage was performed. The resultant list of patient identifiers was used to query and retrieve relevant scans from the hospitals’ Picture archiving and communication system (PACS) servers. Each scan was anonymized and manually checked by the authors to ensure ground truth. The final dataset consisted of 399 unique patient volumetric CT brain scans representing approximately 12,000 images, summarized by the different classes in Table 1. These scans had varying numbers of image slices and slice thickness, owing to variability in CT scanner model and scanning protocols. We prepare the data for 5 five-fold cross-validation. Training, validation, and test CT scan images were augmented eight-fold by flipping along the vertical axis, and rotation up to 45 degrees.

### 2.4. Network Architecture

**Figure 2:**
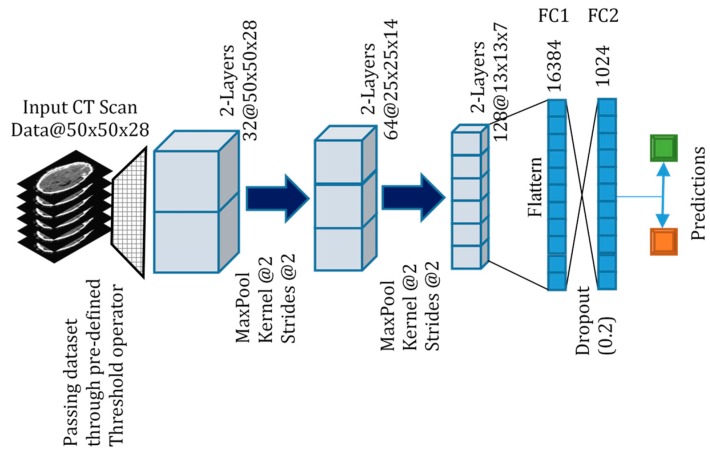
Proposed architecture for binary and multi-class classification of CT scans. The features are visualized using 3D deconvolution visualization methods at each pooling layer.

Table 2 shows the model architecture used in our 3D CNN. We experimented with various model architectures including VGGNet and GoogLeNet, to optimize for the trade-off between classification accuracy and computational time. We aimed to have a model with a straightforward design for easy trouble-shooting and to facilitate real-world implementation. Like Dou et al. [20], we were concerned with the impact of processing large files of volumetric brain images on computation time. However, while they opted for a two-part ensembled screening and discrimination stages, we opted for a single throughput architecture for simplicity and performance. Figure 2 is a pictorial diagram of our proposed 3D CNN. After the necessary preprocessing steps, input volumetric data of 3D CT scans are passed through a pre-defined threshold operator as discussed in Section 2.2 and becomes the input to the 3D CNN.

### 2.5. Training and Implementation

Training was performed on a computer with two Intel Xeon E5-2630 CPU processors, four NVIDIA GTX 1080 Ti Graphical processing units, and 128 GB of DDR4 Random access memory. The project was implemented using the Python programming language and the Google Tensorflow library. We used the rectified linear unit (ReLu) as the activation function, the Adam optimizer, and cross-entropy as the loss function. We used the grid search approach for optimizing the learning rate, dropout, and kernel size of the convolution and pooling layers. We varied learning rates from (0.1, 0.001, 0.0001), and dropout from (0, 0.1, 0.2, 0.3, 0.4, 0.5, 0.6) after each convolution layer and fully connected layer. The kernel sizes of convolution layers were varied from (1 × 1 × 1, 2 × 2 × 2, 3 × 3 × 3, 5 × 5 × 5), and pooling kernel sizes ranged from (1 × 1 × 1, 2 × 2 × 2). Eventually, we set the learning rate at 0.001 and set the dropout after the fully connected layer at 0.2. We employed a convolutional kernel size of 3 × 3 × 3 at each layer, with a 2 × 2 × 2 pooling kernel after each convolutional layer. We set β1 = 0.001, β2 = 0.999; ε = 10^−8^.

#### Metrics

To evaluate our network performance, we measured the Sensitivity (S), Precision (P) and F_1_ scores across each classification task. TP, FP, FN refer to true positive, false positive, and false negative, respectively. The F_1_ score is defined as the harmonic average of Sensitivity and Precision and is a measure of a test’s accuracy. An F_1_ score of 1 indicates perfect Sensitivity and Precision, while a score of 0 indicates the opposite.

(5) $$\begin{array}{c} S=\frac{TP}{TP+FN},\;\;S=\frac{TP}{TP+FP} \end{array}$$

(6) $$\begin{array}{c} F_{1}=\frac{2}{\frac{1}{S}+\frac{1}{P}} \end{array}$$

The confusion matrix for this 4 Class classification problem is tabulated in Table 3. Table 4 summarizes the Sensitivity, Precision, and F_1_ scores, re-casting the multi-class problem into a 2-class problem (Normal versus a specific class) to calculate these metrics.

## 3. Results

We performed experiments involving binary classification (Normal versus SAH, IPH, ASDH, BPH) and multi-class classification. In the latter, BPH was left out as BPH contains features of Normal, SAH, IPH, and ASDH. For the binary classification experiments, we implemented the experiments with and without the thresholding method described in Section 2.2.

For the multi-class classification experiments, the model discriminated between four classes with an overall F1 score of 0.684. Table 3 presents the confusion matrix for the multi-class classification. The actual number for each class represents the augmented test set after the original test set was augmented eight-fold. The respective F1 scores for each class are Normal: 0.819, SAH: 0.639, IPH: 0.427, ASDH: 0.829. Thresholding was not implemented for the multi-class classification as it is optimized for binary classification. The model performed well for Normal and ASDH scans, but only moderately well for SAH. Interestingly, although the training dataset for ASDH was the smallest, the model was able to discriminate this the best. This may be due to the fact that ASDH images are visually grossly asymmetrical compared to the other classes. This is due to brain compression from the significant subdural hemorrhage (see Figure 1), and may be a strongly activated discriminatory feature. Surprisingly, a significant number of IPH scans were misinterpreted as SAH, and we hypothesize that this may be due to subtle SAH traces that may appear on some of the IPH scans.

Table 4 summarizes the results from the 2-class classification experiments. Overall, our model was able to discriminate between normal and each of the classes with satisfactory results, matching or exceeding previously published results for similar work (Table 5). We demonstrate that for every class, the implementation of the thresholding technique improves all the evaluation metrics. The largest increase was seen in the ASDH class, where the F1 score increased from 0.706 to 0.952, despite a small training set. Various model architectures, input image, and filter sizes were modified to optimize for accuracy and computational time. The original image size of the CT scans was 512 × 512 × 28 pixels (at 5 mm slice thickness), and the input size to our model was 50 × 50 × 28 pixels. Further decreasing the input size resulted in deteriorating model performance. We posit that the thresholding technique improves the signal-to-noise ratio for each input image by downplaying the extraneous image features, thereby accentuating the presence of acute hemorrhage. We also found that the thresholding technique decreased training time for the respective models significantly. For example, in the Normal versus SAH classification task, the model training time was decreased from over 10 h to 1 h 32 min. 3D CNNs are computationally intensive to train, and any decrease in computational cost and time is advantageous in clinical deployment.

## 4. Discussion

Acute brain hemorrhage is a common neurosurgical emergency which can result in severe patient morbidity and mortality. It is the result of myriad causes, including trauma, hypertension, cerebral aneurysm rupture, and the treatment of each is different. Depending on the clinical condition, the patient may require close observation in a high dependency or intensive care setting, or immediate neurosurgical operation. It is imperative to minimize the time from diagnosis to treatment, to give the patient the best chance of recovery.

We propose an automated 3D CNN to classify volumetric CT brain data into various hemorrhagic variants, to assist doctors in expediting patient treatment. We trained and tested our model on 399 CT brain images from our hospital to classify CT brain scans into normal, SAH, IPH, ASDH and BPH. These classes were chosen as the neurosurgical treatment for each class is different.

**Figure 3:**
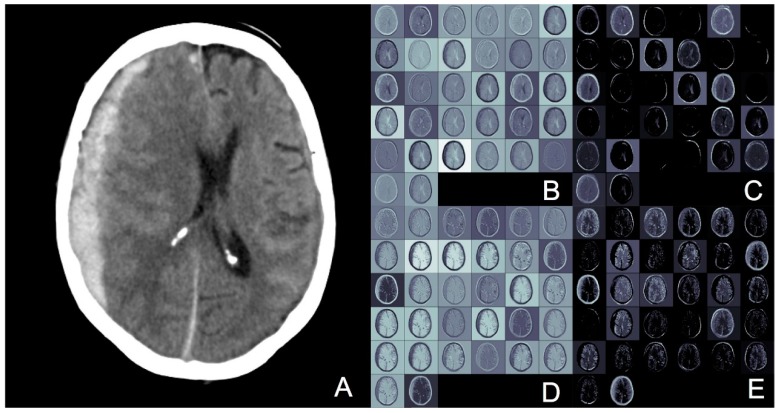
To demonstrate the effect of thresholding, a single slice of an ASDH CT Brain scan is shown, with the corresponding activations of convolution and pooling layers. **A**, original image. **B**, 1st convolution layer. **C**, 1st pooling layer. **D**, 1st convolution layer with thresholding applied. **E**, 1st pooling layer with thresholding applied. D and E appear sharper than B and C, demonstrating how thresholding can accentuate abnormal areas, and improve classifier performance.

We also proposed and implemented a novel pixel thresholding method to detect acute hemorrhage on CT brain scans. This method improved classifier performance on our dataset and can be conceivably exported for use in other datasets for other anatomical regions, where acute hemorrhage detection is required. Potentially, aside from detecting acute blood, this method can also be generalized for the detection of other abnormalities such as tumors. Figure 3 demonstrates an image slice of a CT brain with acute subdural hemorrhage. The hemorrhage is the white crescent on the left of the image, which is putting pressure on the grey areas of brain, and pushing it to the right of the image. To visualize the activations in the 3D CNN better, we used the deconvolution technique described by Yosinski et al. [25] to visualize this single image. The top row of the image (boxes B and C) represent convolution layer 1 and pooling layer 1, respectively. The bottom row (boxes D and E) represent the same layers with thresholding applied. The difference with thresholding is that the edges of the target hemorrhagic lesion appear more distinct and sharper, which may account for why thresholding improves classification accuracy.

In this paper, we demonstrate state-of-the-art 3D CNN classifier performance for different classes of acute brain hemorrhage. In addition, the application of our thresholding method for acute hemorrhage enables our model to achieve further additional improvement in classification. In the 4-class classification task, our model achieved an overall F1 score of 0.684. To our knowledge, there has been no published work involving multi-class classification of different classes of brain hemorrhage for comparison. In the 2-class classification task, the best performance was achieved in differentiating normal from ASDH scans, with a F1 score of 0.952 using our proposed thresholding method. The largest improvement with thresholding was also seen in the ASDH class, as the initial F1 score was only 0.706.

There has been a long history in the attempt to analyze hemorrhage in volumetric CT and MRI data. Before the widespread use of CNNs in image analysis, methods used included Hopfield networks [26], support vector machines [27], and segmentation masking with logistic classifiers [28]. Although these simple classifiers performed well, they were often applied to single image slices with obvious pathology, that were often manually chosen, which, therefore, represents an unrealistic problem scenario. Hybrid 2D CNN methods exemplified by Roth et al. [14] were a bridge to the 3D CNN training of networks. Fully 3D CNN model local and contextual spatial dependencies and extract features in all three dimensions of image voxels. Kamnitsas et al. [16] exploited the dense inference technique and small kernels, to segment lesional areas on brain MRI scans. Of note, they used a dual 11-layer 3D CNN pathway to process images at multiple scales, and Conditional Random Fields to decrease their false positive rate.

There are two points worth noting in our network architecture design. First, we deliberately kept our network architecture simple with relatively few layers, and did not leverage on other techniques, such as ensembling and transfer-learning. This was for both practical and theoretical considerations. The application of full 3D CNN to volumetric medical images is nascent, and by purposely keeping the model architecture straightforward, we are able to assess the effect size of various hyperparameter-tuning quickly, experiment with various architectures efficiently, and to troubleshoot network errors expediently. Running 3D CNN is computationally intensive, and a simpler network mitigates lengthy run-times.

From a more theoretical perspective, a straightforward architecture also allows us to grasp a sense of the baseline performance of a 3D CNN, and to describe our experimentations with a clear mathematical description. We believe that a firm theoretical framework will help in directing further areas for exploration, rather than blindly tuning hyperparameters, or implementing network boosting ensembling architectures without understanding.

The second point is that with a straightforward network architecture design, we were able to demonstrate the clear improvement that our thresholding method brought for detecting acute hemorrhage. We arrived at this thresholding method by observing radiologists as they scrolled through various patient CT scans. We noted that in situations where areas of acute brain hemorrhage were subtle, the radiologists would increase and decrease the image contrast to accentuate the appearance of the abnormality, which in this case was blood. We did the same thing while studying our dataset, and, therefore, explored how this optimization of human visual cognition or analysis could be implemented algorithmically. We took inspiration from the work of Zhang et al. [23] who used pixel intensity ’spatial histograms’ for object detection within an image. Our underlying assumption was similar, that objects of a certain class would have similar patterns of pixel intensities. Where we differ is that while Zhang et al. were concerned with the spatial location of the object, we are concerned more with the actual presence of a particular pattern of pixel intensity, and the point of divergence from the pattern denoting a normal scan. This is because in CT brain scans depicting a hemorrhage, the blood even within the same class of brain hemorrhage can appear in several different areas of the brain.

The proposed architecture in this work has important clinical significance. The different abnormal diagnoses studied in this work all exert a significant epidemiological and social-economic toll on healthcare systems and societies. SAH, IPH, ASDH, and BPH are all neurosurgical emergencies that require immediate but different treatments to maximize the likelihood of patient survival and to achieve a good long-term functional outcome. Our work has a role in helping clinicians minimize the time between diagnosis and treatment, especially in hospitals that may not have a radiologist after hours, or in remote rural settings where no clinicians are available. Even in large tertiary care hospitals with 24 h-radiologists, our proposed architecture framework can assist radiologists by triaging important abnormal scans from the large numbers of normal scans that are read sequentially as patients are scanned.

One limitation of this work is the relatively small and un-balanced dataset that we worked with, which is a common issue in medical image analysis, with a bias towards normal samples. At 399 images, our dataset size is comparable to those used in many other works [20,24] but smaller than the almost 40,000 CT scans used by Jnawali et al. [19]. Despite this, data augmentation techniques have resulted in performance comparable to Jnawali et al.’s much larger dataset. This may be due to the fact that medical data is relatively homogeneous in appearance, compared to natural image processing tasks. It would be interesting to study what is the optimal dataset size for processing specific volumetric image classification tasks, in CT, MRI, 3D ultrasound images for different lesions. We addressed concerns of overfitting with known mitigating techniques, such as dropout, which were applied to the early layers in our network.

There are many avenues for further exploration in volumetric medical image analysis. CNNs and 3D CNNs have been the dominant network architecture in image analysis, but unsupervised learning methods for medical image analysis are emerging for 3D object generation [29,30], and they have been largely unexplored in the context of 3D medical image analysis. Generative architectures, such as variational autoencoders and generative adversarial networks, have not been applied to volumetric medical data, and these techniques may potentially mitigate the need for large well-labelled datasets. Specific to our work, we intend to explore if adding a screening stage to a 3D CNN, or multi-scale receptive fields can improve performance, as some authors have demonstrated [16,20] for MRI brain scans. The addition of a memory or attention-based component to model long term dependencies in 3D medical image analysis [24,31,32] is also interesting for further investigation, as there is evidence for a strong biological correlate with the mammalian visual system [33].

## 5. Conclusions

This work presented the implementation of a 3D CNN to classify and diagnose volumetric CT brain data. Normal CT brains and a variety of abnormal scans constituting different types of brain hemorrhage were classified by our 3D CNN. We also implement a novel optimization method to detect acute hemorrhage on CT scans. The proposed 3D CNN can automatically detect important normal and abnormal features of cerebral anatomy without handcrafting, or significant data pre-processing. Computational costs were also modest, which will add to straightforward implementation. Results from classification experiments demonstrated that the 3D CNN outperforms previously published methods in detecting abnormal brain scans with hemorrhage, with higher sensitivity and recall. Our 3D CNN can be applied to other volumetric medical data and can be used to expedite and improve patient care.

## Figures and Tables

**Table 1:** Number of original unique patient computed tomography (CT) scans.

Normal	Subarachnoid Hemorrhage (SAH)	Intraparenchymal Hemorrhage (IPH)	Subdural Hemorrhage (ASDH)	Brain Polytrauma Hemorrhage (BPH)
130	141	61	32	35

**Table 2:** Model architecture of the 3-dimensional convolutional neural networks (3D CNN) used in this work.

Layer	Kernel Size	Stride	Output Size (Width × Length × Depth × Filters)
Input	-	-	50 × 50 × 28
Convolution 1	3 × 3 × 3	1	50 × 50 × 28 × 32
Pooling 1	2 × 2 × 2	2	25 × 25 × 14 × 32
Convolution 2	3 × 3 × 3	1	25 × 25 × 14 × 64
Pooling 2	2 × 2 × 2	2	13 × 13 × 7 × 64
Convolution 3	3 × 3 × 3	1	13 × 13 × 7 × 128
Pooling 3	2 × 2 × 2	2	7 × 7 × 4 × 128
Fully Connected 1	-	-	25,088 × 1024
Fully Connected 2	-	-	1024 × 2

**Table 3:** Multi-Class Classification for Normal and Abnormal CT Scans.

Actual					
		Normal	SAH	IPH	ASDH
**Predicted**	**Normal**	129	30	4	0
	**SAH**	7	100	35	3
	**IPH**	15	31	32	0
	**ASDH**	1	7	1	29

**Table 4:** 2-Class Classification Results (Normal versus a specific Abnormal Class).

Task	Sensitivity	Precision	F1 Score	AUC
Normal versus SAH	0.947	0.818	0.878	0.900
(no thresholding)				
Normal versus SAH	1.000	0.864	0.927	0.950
(with thresholding)				
Normal versus IPH	0.819	0.881	0.849	0.958
(no thresholding)				
Normal versus IPH	0.944	0.919	0.932	0.989
(with thresholding)				
Normal versus ASDH	0.750	0.666	0.706	0.953
(no thresholding)				
Normal versus ASDH	0.938	0.968	**0.952**	0.999
(with thresholding)				
Normal versus BPH	0.925	0.881	**0.902**	0.989
(no thresholding)				
Normal versus BPH	0.850	1.00	0.919	0.990
(with thresholding)				

Highest F1 scores are in bold.

**Table 5:** Comparison of results involving brain hemorrhage detection in volumetric brain scans.

Task	Reference	Sensitivity Precision F1	Task	Reference
Detecting cerebral micro-bleeds	Dou et al. [20]	93.2 %	44.3 %	-
on MRI brain scans				
Brain hemorrhage classification	Grewal et al. [24]	88.6 %	81.3 %	0.85
on CT scan				
Brain hemorrhage classification	Jnawali et al. [19]	77.0 %	87.0 %	0.83
on CT scan				
Brain hemorrhage classification	Our best performing	92.5 %	88.1 %	0.90
on CT scan	method (no thresholding)			
Brain hemorrhage classification	Our best performing	93.8 %	96.8 %	0.95
on CT scan	method (with thresholding)			

Dou et al. expressed their evaluation metric as Sensitivity, Precision, and False positives per subject.
